# Genome Profiling for Aflatoxin B_1_ Resistance in *Saccharomyces cerevisiae* Reveals a Role for the CSM2/SHU Complex in Tolerance of Aflatoxin B_1_-Associated DNA Damage

**DOI:** 10.1534/g3.120.401723

**Published:** 2020-09-29

**Authors:** Nick St. John, Julian Freedland, Henri Baldino, Francis Doyle, Cinzia Cera, Thomas Begley, Michael Fasullo

**Affiliations:** * College of Nanoscale Science and Engineering, State University of New York Polytechnic Institute, Albany, New York 12203; † RNA Institute, University at Albany, Albany, New York 12222

**Keywords:** genome profiling, aflatoxin, budding yeast, DNA damage, postreplication repair

## Abstract

Exposure to the mycotoxin aflatoxin B1 (AFB_1_) strongly correlates with hepatocellular carcinoma (HCC). P450 enzymes convert AFB_1_ into a highly reactive epoxide that forms unstable 8,9-dihydro-8-(*N*7-guanyl)-9-hydroxyaflatoxin B1 (AFB_1_-*N*^7^-Gua) DNA adducts, which convert to stable mutagenic AFB_1_ formamidopyrimidine (FAPY) DNA adducts. In CYP1A2-expressing budding yeast, AFB_1_ is a weak mutagen but a potent recombinagen. However, few genes have been identified that confer AFB_1_ resistance. Here, we profiled the yeast genome for AFB_1_ resistance. We introduced the human CYP1A2 into ∼90% of the diploid deletion library, and pooled samples from CYP1A2-expressing libraries and the original library were exposed to 50 μM AFB_1_ for 20 hs. By using next generation sequencing (NGS) to count molecular barcodes, we initially identified 86 genes from the CYP1A2-expressing libraries, of which 79 were confirmed to confer AFB_1_ resistance. While functionally diverse genes, including those that function in proteolysis, actin reorganization, and tRNA modification, were identified, those that function in postreplication DNA repair and encode proteins that bind to DNA damage were over-represented, compared to the yeast genome, at large. DNA metabolism genes also included those functioning in checkpoint recovery and replication fork maintenance, emphasizing the potency of the mycotoxin to trigger replication stress. Among genes involved in postreplication repair, we observed that *CSM2*, a member of the *CSM2(SHU)* complex, functioned in AFB_1_-associated sister chromatid recombination while suppressing AFB_1_-associated mutations. These studies thus broaden the number of AFB_1_ resistance genes and have elucidated a mechanism of error-free bypass of AFB_1_-associated DNA adducts.

The mycotoxin aflatoxin B1 (AFB_1_) is a potent hepatocarcinogen. The signature p53 mutation, p53-Ser249, is often present in liver cancer cells from hepatocellular carcinoma (HCC) patients from AFB_1_-exposed areas, suggesting that AFB_1_ is a potent carcinogen because it is a genotoxin (Hsu *et al.* 1991; Shen and Ong 1996). A mutagenic signature associated with AFB_1_ exposure has been identified in HCC (Chawanthayatham *et al.* 2017; Huang *et al.* 2017). However, AFB_1_ is not genotoxic *per se* but is metabolically activated by P450 enzymes, such as CYP1A2 and CYP3A4 (Crespi *et al.* 1991; Eaton and Gallagher 1994; Gallagher *et al.* 1996), to form a highly reactive AFB_1_-8-9-epoxide (Baertschi *et al.* 1988). The epoxide reacts with protein, RNA, and DNA, yielding the unstable 8,9-dihydro-8-(*N*7-guanyl)-9-hydroxyaflatoxin B1 (AFB1-*N*^7^-Gua) adducts that convert into stable AFB_1_-formamidopyrimidine (FAPY) adducts (Essigmann *et al.* 1977; Lin *et al.* 1977; Croy and Wogan 1981). The anomers of the AFB_1_-FAPY-DNA adduct block DNA replication or cause mutations in *Escherichia coli* (Smela *et al.* 2002; Brown *et al.* 2006) and *in vitro* (Lin *et al.* 2014). Metabolically active AFB_1_ can also indirectly damage DNA through oxidative stress (Shen *et al.* 1995; Beddard and Masey 2006; Bernabucci *et al.* 2011; Singh *et al.* 2015). Identifying genes that repair AFB_1_-associated DNA damage could help identify which individuals are at elevated risk for HCC. However, epidemiological data has been inconsistent, and only a few candidate DNA repair genes have been proposed, such as XRCC1 (Pan *et al.* 2011, Xu *et al.* 2015), XRCC3 (Long *et al.* 2008; De Mattia *et al.* 2017) and XRCC4 (Long *et al.* 2013).

AFB_1_ resistance genes have been identified from model organisms, revealing mechanisms by which AFB_1_-associated DNA adducts can be both tolerated and excised. Both prokaryotic and eukaryotic nucleotide excision repair (NER) pathways function to remove AFB_1_-associated DNA adducts (Leadon *et al.* 1981; Alekseyev *et al.* 2004; Bedard and Massey 2006). Recently, the base excision repair gene (BER) NEIL1 has been implicated in direct repair AFB_1_-associated DNA adducts (Vartanian *et al.* 2017). To tolerate persistent AFB_1_-associated DNA lesions, translesion polymerases in yeast, such as those encoded by *REV1* and *REV7*, and in mouse, such as Polζ, confer resistance and may promote genome stability (Lin *et al.* 2014).

While yeast does not contain endogenous P450 genes that can metabolically activate AFB_1_ into an active genotoxin (Sengstag *et al.* 1996; Van Leeuwen *et al.* 2012, Fasullo *et al.* 2014), human CYP1A2 can be expressed in yeast cells (Sengstag *et al.* 1996; Fasullo *et al.* 2014). Interestingly, metabolically activated AFB_1_ is a potent recombinagen but weak mutagen (Sengstag *et al.* 1996). CYP1A2-activated AFB_1_ reacts to form both the unstable AFB_1_-N^7^-Gua adducts and the stable AFB_1_-FAPY DNA adducts (Fasullo *et al.* 2008). The AFB_1_-associated DNA damage, in turn, triggers a robust DNA damage response that includes checkpoint activation (Fasullo *et al.* 2008), cell cycle delay (Fasullo *et al.* 2010), and the transcriptional induction of stress-induced genes (Keller-Seitz *et al.* 2004; Guo *et al.* 2006). Profiles of the transcriptional response to AFB_1_ exposure reveals induction of genes in growth and checkpoint signaling pathways, DNA and RNA metabolism, and protein trafficking (Keller-Seitz *et al.* 2004; Guo *et al.* 2006). While genes involved in recombinational repair and postreplication repair confer AFB_1_ resistance (Keller-Seitz *et al.* 2004; Guo *et al.* 2005; Fasullo *et al.* 2010), it is unclear the functional significance of many genes in the stress induced pathways in conferring resistance since transcriptional induction is not synonymous with conferring resistance (Birrell *et al.* 2002).

In this study, we profiled the yeast genome for AFB_1_ resistance. We asked which genes confer AFB_1_ resistance in the presence or absence of human CYP1A2 expression by screening the non-essential diploid collection by high throughput sequencing of molecular barcodes (Pierce *et al.* 2006; St Onge *et al.* 2007; Smith *et al.* 2010). While we expected to identify NER, recombinational repair, and postreplication repair, which had previously been identified (Keller-Seitz *et al.* 2004; Guo *et al.* 2005; Fasullo *et al.* 2010), our high throughput screen identified novel genes involved in AFB_1_ resistance. These included genes involved in Rad51 assembly, cell cycle progression, RNA metabolism, and oxidative stress. Our results thus underscore the importance of recombination in both mutation avoidance and in conferring AFB_1_ resistance.

## Materials and Methods

### Strains and plasmids

Yeast strains were derived from BY4741, BY4743 (Brachmann *et al.* 1998) or YB204 (Dong and Fasullo 2003); all of which are of the S288C background (Table S1). The BY4743 genotype is *MAT*a/α *his3*Δ*1/**his3*Δ*1 **leu2*Δ*0/**leu2*Δ*0 **LYS2**/**lys2*Δ*0 **met15*Δ*0/**MET15* *ura3*Δ*0/**ura3*Δ*0*. The diploid and haploid homozygous deletion libraries were purchased from Open Biosystems, and are now available from Dharmacon (http://dharmacon.gelifesciences.com/cdnas-and-orfs/non-mammalian-cdnas-and-orfs/yeast/yeast-knockout-collection/). The pooled diploid homozygous deletion library (n = 4607) was a gift of Chris Vulpe (University of Florida).

To construct the *csm2* *rad4* and *csm2* *rad51* double mutants, we first obtained the haploid *csm2* strain (YA288, Table S1) from the haploid BY4741-derived deletion library. We introduced the *his3* recombination substrates (Fasullo and Davis 1987) to measure unequal sister chromatid exchange (SCE) in the *csm2* mutant by isolating the meiotic segregant YB558 from a diploid cross of YB204 with YA288. This *csm2* strain (YB558) was subsequently crossed with *MAT*α *rad4*::*NatMX* (YA289) and the *csm2*::*KanMX **rad4*::*NatMX* meiotic segregant (YB660) was obtained. The *rad51* *csm2* double mutant was made by one step gene disruption (Rothstein 1983) using the *Bam*^1^H fragment *rad51*Δ (Shinohara *et al.* 1992) to select for Ura^+^ transformants in YB558.

Using LiAc-mediated gene transformation we introduced human CYP1A2 into BY4741, *csm2*, *rad4*, *rad51*, *csm2* *rad51*, and *csm2* *rad4* strains. The CYP1A2-expression plasmid, pCS316, was obtained by CsCl centrifugation (Ausubel *et al.* 1995) and the restriction map was verified based on the nucleotide sequence of the entire plasmid. An alternative CYP1A2-expression plasmid, pCYP1A2_NAT2 was constructed by removing the hOR sequence from pCS316 and replacing it with a *Not*1 fragment containing the human NAT2.

### Media and chemicals

Standard media were used for the culture of yeast and bacterial strains (Burke *et al.* 2000). LB-AMP (Luria broth containing 100 μg/ml ampicillin) was used for the culture of the bacterial strain DH1 strain containing the vector pCS316. Media used for the culture of yeast cells included YPD (yeast extract, peptone, dextrose), SC (synthetic complete, dextrose), SC-HIS (SC lacking histidine), SC-URA (SC lacking uracil), and SC-ARG (SC-lacking arginine). Media to select for canavanine resistance contained SC-ARG (synthetic complete lacking arginine) and 60 μg/mL canavanine (CAN) sulfate, and media to select for 5-fluoroorotic acid (FOA) resistance contained SC-URA supplemented with 4x uracil and FOA (750 μg/ml), as described by Burke *et al.* (2000). FOA plates contained 2.2% agar; all other plates contained 2% agar. AFB_1_ was purchased from Sigma Co., and a 10 mM solution was made in dimethyl sulfoxide (DMSO).

### Measuring DNA Damage-Associated recombination and mutation events

To measure AFB_1_-associated genotoxic events, log phase yeast cells (*A*_600_ = 0.5-1) were exposed to indicated doses of AFB_1_, previously dissolved in DMSO. Cells were maintained in synthetic medium (SC-URA) during the carcinogen exposure. After the exposure, cells were washed twice in H_2_O, and then plated on SC-HIS or SC-ARG CAN to measure unequal SCE or mutation frequency, respectively. An appropriate dilution was inoculated on YPD to measure viability (Fasullo *et al.* 2008).

### Construction of CYP1A2-expression library

To introduce CYP1A2 (pCS316, Sengstag *et al.* 1996, and pCYP1A2_NAT2) into the yeast diploid deletion collection, we used a modified protocol for high throughput yeast transformation in 96-well plates (Gietz and Schiestl 2007). In brief, FOA^R^ isolates were isolated from each individual strain in the diploid collection and inoculated in 96-well plates, each containing 100 μl of YPD medium. After incubation over-night at 30°, plates were centrifuged, washed in sterile H_2_O, and resuspended in one-step buffer (0.2 N LiAc, 100 mM DTT, 50% PEG, MW 3300, 500 μg/ml denatured salmon sperm DNA). After addition of 1 μg pCS316 and incubation for 30 min at 30°, 10 μl were directly inoculated on duplicate SC-URA plates. Two Ura^+^ transformants were chosen corresponding to each well and frozen in SC-URA 0.75% DMSO. We introduced the CYP1A2-containing plasmids into approximately 90% of the deletion collection.

### Functional profiling of the yeast genome

The CYP1A2-expressing libraries were pooled and frozen in SC-URA medium containing 0.75% DMSO (n = 4150). The pooled cells (100 μl) were added to 2 ml of SC-URA and allowed to recover for two hours. Cell were then diluted to A_600_ = 0.85 in 2 ml of SC-URA and exposed to either 50 μM AFB_1_ in 0.5% DMSO, and 0.5% DMSO alone. Cells were then incubated with agitation at 30° for 20 hs. Similarly, the pooled BY4743 library (n = 4607) was directly diluted to A_600_ = 0.85 in YPD and also exposed to 50 μM AFB_1_ and DMSO for 20 hs. Independent triplicate experiments were performed for each library and each chemical treatment.

After AFB_1_ exposure, cells were washed twice in sterile H_2_O and frozen at -80°. Cells were resuspended in 10 mM Tris-HCl, 1 mM EDTA, 100 mM NaCl, 2% Triton X-100, 1% SDS, pH 8 and DNA was isolated by “smash and grab (Hoffman and Winston 1987).” Barcode sequences, which are unique for each strain in the deletion collection (Giaever *et al.* 2002; Giaever *et al.* 2004), were amplified by PCR using a protocol described by Smith *et al.* (2010). The primers used for amplification are listed in the Table S2. 125 bp PCR products were then isolated from 10% polyacrylamide gels by diffusion in 0.5M NH_4_Ac 1 mM EDTA for 24 hs (30°) followed by ethanol precipitation. The DNA was quantified after being resuspended in Tris EDTA pH 7.5 and the integrity of the DNA was verified by electrophoresis on 10% polyacrylamide. Equal amounts of DNA were pooled from treated and untreated samples. The uptags were then sequenced using the Illumina Platform at the University Buffalo Genomics and Bioinformatics Core (Buffalo, New York). Sequence information was then uploaded to an accessible computer server for further analysis. The software to demultiplex the sequence information, match the uptag sequences with the publish ORFs, and calculate the statistical significance of the differences in log2N ratios was provided by F. Doyle. Tag counts were analyzed with the TCC Bioconductor package (Sun *et al.* 2013) using TMM normalization (Robinson and Oshlack 2010) and the edgeR test method (Robinson *et al.* 2010). Statistical testing was performed with edgeR TCC package for tag count comparison in the R programming language; an R script with invocation details is provided in the File S1. Data files have been deposited in the Gene Expression Omnibus database, GSE129699.

### Over-enrichment analysis

Gene Ontology (GO) categories were identified by a hypergeometric distribution with freely available software from Princeton University using the Generic Gene Ontology Term Finder (http://go.princeton.edu/cgibin/GOTermFinder) and Bonferroni correction for *P* values. Enriched GO terms were further refined using ReViGO (Supek *et al.* 2011). Enrichment analysis was analyzed using Panther (http://pantherdb.org/tools/) with a P value cutoff of < 0.05 (Cherry *et al.* 2012, Mi *et al.* 2016). The AFB_1_ sensitivity of mutants corresponding to GO groups was verified by growth curves and trypan blue assays.

### Growth assays in 96 well plate to measure AFB_1_ sensitivity

In brief, individual saturated cultures were prepared for each yeast strain. Cell density was adjusted to ∼0.8 × 10^7^ cells/ml for all cultures. We maintained the cells in selective medium (SC-URA). In each microtiter well, 95 μl of media and 5 μl of cells (8 × 10^4^ cells) were aliquoted in duplicate for blank, control and experimental samples. For experimental samples, we added AFB_1_, dissolved in DMSO, for a final concentration of 50 μM and 100 μM. The microtiter dish was placed in a plate reader that is capable of both agitating and incubating the plate at 30°, as previously described (Fasullo *et al.* 2010; Fasullo *et al.* 2014). We measured the A_600_ at 10 min intervals, for a total period for 24 hs, 145 readings. Data at 1h intervals was then plotted. To avoid evaporation during the incubation, the microtiter dishes were sealed with clear optical tape (Fasullo *et al.* 2010). To calculate area under the curve (AUC), we used a free graphing application (https://www.padowan.dk/download/), and measured the time interval between 0-20 hs, as performed in previous publications (O’Connor *et al.* 2012). After cells were exposed to AFB_1_ and DMSO, we calculated the ratio (AUC _AFB1_/AUC_DMSO_) × 100% to determine the percent yeast growth obtained in the presence of the toxin. For the wild-type diploid BY4743 expressing CYP1A2 (YB556), the percent of yeast growth after exposure to 50 μM is 89.7 ± 2.5. Statistical significance of differences between growth percentages for diploid strains and BY4743 were determined by the Student’s *t*-test, assuming constant variance between samples.

To determine epistasis of AFB_1_-resistance genes, we calculated the deviation ε according to ε = W_xy_- W_x_ x W_y_, where W_x_ and W_y_ are the fitness coefficients determined for each single mutant exposed to AFB_1_ and W_xy_ is the product. Fitness was calculated by determining the generation time of both the single and double mutants over three doubling times. Zero and negative values are indicative of genes that do not interact or participate in the same pathway to confer fitness (St. Onge *et al.* 2007).

### Trypan blue exclusion assay to monitor cell viability after acute AFB_1_ exposure

To measure cell viability after AFB_1_ exposure, we performed a trypan blue exclusion assay. Selected strains expressing CYP1A2 were inoculated in SC-URA until cultures reached an A_600_ ∼0.1-0.5, and then exposed to either 50 μM in AFB_1_ or DMSO (solvent) alone. After incubating for 3 hs, cells were washed twice in sterile phosphate buffered saline (PBS) and stained with trypan blue at a final concentration ∼10 μg/ml (Liesche *et al.* 2015). Cells were counted in a Nexcelom cellometer T4, according to the manufacturer’s instructions. A minimum of 10^4^ cells were counted and all strains were tested at least twice. Statistical significance was determined by the Student’s *t*-test.

### Western blot analysis

Expression of CYP1A2 was determined by Western blots and MROD assays. Cells were inoculated in SC-URA medium. Cells in log growth phase (A_600_ = 0.5–1) were concentrated and protein extracts were prepared as previously described by Foiani *et al.* (1994). Proteins were separated on 10% acrylamide/0.266% bis-acrylamide gels and transferred to nitrocellulose membranes. Human CYP1A2 was detected by Western blots using goat anti-CYP1A2 (Abcam), and a secondary bovine anti-goat antibody. For a loading control on Western blots, β-actin was detected using a mouse anti-β-actin antibody (Abcam 8224) and a secondary goat anti-mouse antibody. Signal was detected by chemiluminescence, (Fasullo *et al.* 2014).

### Measuring CYP1A2 enzymatic activity

We measured CYP1A2 enzymatic activity using a modified protocol described by Pompon *et al.* (1996). In brief, cells obtained from 100 ml of selective media were pelleted and resuspended in 5 ml Tris EDTA KCl (pH 7.5, TEK) buffer. After five minute incubation at room temperature, cells were pelleted, resuspended in 1 ml 0.6 M Sorbitol Tris pH 7.5, and glass beads were added. Cells were lysed by agitation. The debris was pelleted at 10,000 × g at 4°, and the supernatant was diluted in 0.6 M Sorbitol Tris pH 7.5 and made 0.15 M in NaCl and 1% in polyethylene glycol (MW 3350) in a total volume of 5 ml. After incubation on ice for 1 hr. and centrifugation at 10,000 rpm for 20 min, the precipitate was resuspended in Tris 10% glycerol pH 7.5, and stored at ^−^80°.

CYP1A2 enzymatic activity was measured in cell lysates by quantifying 7-methoxyresorufin O-demethylase (MROD) activities (Fasullo *et al.* 2014), using a protocol similar to that quantifying ethoxyresorufin O-deethylase (EROD) activity (Eugster *et al.* 1992; Sengstag *et al.* 1994). The buffer contained 10 mM Tris pH 7.4, 5μM methoxyresorufin (Sigma) and 500 μM NADPH. The production of resorufin was measured in real-time by fluorescence in a Tecan plate reader, calibrated at 535 nm for excitation and 580 nm for absorption, and standardized using serial dilutions of resorufin. The reaction was started by the addition of NADPH and resorufin was measured at one minute intervals during the one hour incubation at 37°; rat liver microsomes (S9) were used as a positive control while the reaction without NADPH served as the negative control. Enzyme activities were measured in duplicate for at least two independent lysates from each strain and expressed in pmol/min/mg protein.

### Data availability

All yeast strains and plasmids are available upon request and are detailed in Table S1. Three supplementary tables and two supplementary figures have been deposited in figshare. Next generation sequencing data (NGS) of barcodes are available at GEO (https://www.ncbi.nlm.nih.gov/geo/query/acc.cgi?acc=GSE129699). Additional supplementary files include six supplementary tables, three supplementary figures, and one file. Table S1 is a complete listing of strains and their genotypes. Table S2 is a complete listing of DNA oligonucleotides used in the HiSeq2000 experiments. Table S3 lists the methoxyresorufin demethylase (MROD) activities obtained from microsomal extracts of four deletion strains. Table S4 lists strains that were associated with positive m values. Table S5 lists strains that have not yet been confirmed as AFB_1_ sensitive by any criteria. Table S6 is a complete listing of the GO groups according to process for all 86 genes associated with negative m values identified in screens for AFB1 resistance. Figure S1 describes the percentage of growth obtained after 86 strains were exposed to 50 μM AFB_1_ and a subset of the more resistant strains that were exposed to both 50 μM and 100 μM AFB1. Figure S2 is a bar graph detailing the percent viability of strains after an acute exposure 50 μM AFB_1_. Figure S3 contains the growth curves for BY4743, *shu1*, *shu2*, and *psy3* after exposure to 50 μM AFB_1_. File S1 describes edge R script with invocation details. Supplemental material available at figshare: https://doi.org/10.25387/g3.12895313.

## Results

**Figure 1:**
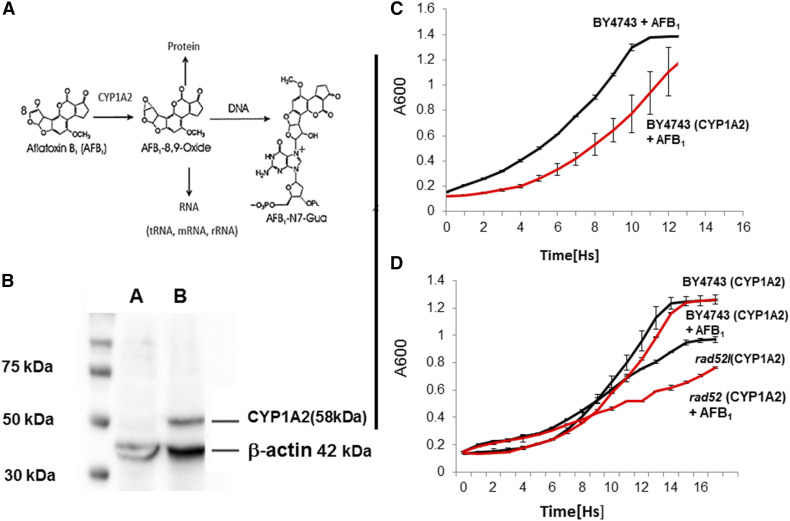
Expression of CYP1A2 in the yeast diploid strain (BY4743). Top left (A) indicates CYP1A2-mediated activation of AFB_1_ to form a highly reactive epoxide that forms DNA, RNA, and protein adduct; Figure 1(A) adapted from Smela *et al*. (2002). The lower left panel (B) is a Western blot indicating 75, 50 and 37 kD molecular weight markers. Lanes A and B are lysates from BY4743 and BY4743 cells expressing CYP1A2 (YB556), respectively. The CYP1A2 (58 kD) protein and the β-actin (42 kD) protein are indicated. Right upper panel (C) is a growth curve of the diploid wild type (BY4743) and YB556 after exposure to 100 μM AFB_1_. Right lower panel (D) is a growth curve of YB556 and *rad52* (CYP1A2, YB665) after exposure to 1% DMSO and 50 μM AFB_1_. Growth (A_600_) is plotted against time (Hs). Standard deviations are indicated at 1 h time points. Red lines indicate strains both expressing CYP1A2 and exposed to AFB_1_.

We used three BY4743-derived libraries to profile the yeast genome for AFB_1_ resistance. The first was a pooled library of 4607 yeast strains, each strain containing a single deletion in a non-essential gene (Jo *et al.* 2009). The second was a pooled library of approximately 4900 strains each containing individual deletions in non-essential genes and was made by introducing pCS316 into each strain by yeast transformation. The third was a pooled library of approximately 5000 strains expressing both CYP1A2 and NAT2; this pooled library can be used to screen polyaromatic and heterocyclic compounds that require CYP1A2 and NAT2 for metabolic activation. By calculating area under the growth curves (AUC) for cells exposed to AFB_1_ and solvent (DMSO) alone and measuring the ratio (AUC_AFB1_/AUC_DMSO_), we determined that the AFB_1_ concentration to elicit ∼10% decrease in growth (D_10,_) for BY4743 expressing CYP1A2 (YB556) was 50 μM, while the dose to elicit a 16% decrease in growth (D_16_) was 100 μM. The number of AFB_1_ -associated DNA adducts formed *in vivo* after exposure to 100 μM AFB_1_ is less than twice of that detected after exposure to 50 μM AFB_1_ (Fasullo *et al.* 2008), suggesting that metabolic activation is more efficient after exposure to 50 μM AFB_1_; we therefore chose 50 μM AFB_1_ exposure to identify genes that confer resistance to AFB_1_-associated metabolites (Figure 1A). The D_10_ for BY4743 expressing CYP1A2 and the human oxidoreductase (hOR) was the same as in BY4743 cells expressing CYP1A2 and NAT2. To confirm metabolic activation of AFB_1_ into a potent genotoxin, we showed that growth of the *rad52* diploid mutant was significantly impaired (Figure 1D). Cells that did not express CYP1A2 showed slight growth delay after cells were exposed to 100 μM AFB_1_ (Figure 1C).

### Confirmation of CYP1A2 activity

To confirm that CYP1A2 was both present and functional in the library, we performed Western blots (Figure 1B) and MROD assays, as in previous studies (Fasullo *et al.* 2014). Two independent assays were performed for four different ORFs (*RAD2*, *RAD18*, *RAD55*, and *OGG1*); the range of average MROD activity was 5-10 units pmol/sec/mg protein (see Table S3). These results are similar to what was observed for the wild type BY4743 expressing CYP1A2 (Fasullo *et al.* 2014) and for various haploid mutants (Guo *et al.* 2005). These studies indicate that CYP1A2 is active in diploid strains and can be detected by Western blots in BY4743-derived strains containing pCS316, in agreement with previous studies (Guo *et al.* 2005).

### Identification of genes by barcode analysis

We classified genes that confer AFB_1_ resistance (q < 0.1) genes into: 1) those that confer resistance to AFB_1_ without CYP activation, and 2) those that confer resistance to P450-activated AFB_1_. After exposing cells to 50 μM AFB_1_, we identified barcodes for approximately 51% and 89% of the genes from pooled deletion library with and without CYP1A2, respectively (for complete listing, see https://www.ncbi.nlm.nih.gov/geo/query/acc.cgi?acc=GSE129699).

One gene, *CTR1*, which functions in high-affinity copper and iron transport (Dancis *et al.* 1994), was identified as statistically different from the pooled library that did not express CYP1A2; this gene was identified twice in the screen (YPR124W and completely overlapping YPR123C). The human homolog confers drug resistance, suggesting human CTR1 is involved in xenobiotic transport (Furukawa *et al.* 2008). No DNA repair genes were identified in screening the library lacking CYP1A2, consistent with observations that cytochrome P450-metabolic activation AFB_1_ is required to form AFB_1_-associated DNA adducts (Fasullo *et al.* 2014).

Using the same stringent assessment (q < 0.1), in three independent screens, we identified 96 ORFs that confer AFB_1_ resistance in cells expressing CYP1A2, of which 86 genes have been ascribed a function, and one ORF, YBR099C, is completely internal to *MMS4*. Genes that confer resistance were associated with a negative m value (Table 1), as defined by fold change on a log2 scale; this indicates that corresponding deletion strains would be relatively depleted after AFB_1_ exposure, compared to exposure to solvent (DMSO) alone. Alternatively, genes associated with a positive m value (Table S4) imply that corresponding mutants would be relatively enriched after AFB_1_ exposure. 43 ORFs were only associated with a positive m value, and nine ORFs were associated with both a negative and positive m values in independent screens (Table S4).

**Table 1:** Fitness scores for 15 AFB_1_ resistant genes related to DNA repair and ranked by significance

Gene*^a^*	m. value*^b^*	Gene Function*^c^*	q.value*^d^*
***RAD54****^#^*	−6.60	DNA-dependent ATPase that stimulates strand exchange; modifies the topology of double-stranded DNA; involved in the recombinational repair of double-strand breaks in DNA; member of the SWI/SNF family of DNA translocases; forms nuclear foci upon DNA replication stress	3.09E-13
*MMS4*	−3.96	Subunit of structure-specific Mms4p-Mus81p endonuclease; cleaves branched DNA; involved in recombination, DNA repair, and joint molecule formation/resolution during meiotic recombination	4.44E-12
RAD2*	−3.73	Single-stranded DNA endonuclease; cleaves single-stranded DNA during nucleotide excision repair to excise damaged	1.03E-10
RAD55**	−3.98	Protein that stimulates strand exchange; stimulates strand exchange by stabilizing the binding of Rad51p to single-stranded DNA	1.96E-07
*REV3*	−4.15	Catalytic subunit of DNA polymerase zeta	2.39E-07
RAD10**	−2.35	Single-stranded DNA endonuclease (with Rad1p); cleaves single-stranded DNA during nucleotide excision repair and double-strand break repair	3.04E-07
*REV1*	−4.07	Deoxycytidyl transferase	4.89E-06
*RAD17*	−4.24	Checkpoint protein; involved in the activation of the DNA damage and meiotic pachytene checkpoints; with Mec3p and Ddc1p, forms a clamp that is loaded onto partial duplex DN	9.13E-06
RAD18**	−3.30	E3 ubiquitin ligase; forms heterodimer with Rad6p to monoubiquitinate PCNA-K164	0.00421
*RAD23*	−6.20	Protein with ubiquitin-like N terminus; subunit of Nuclear Excision Repair Factor 2 (NEF2) with Rad4p that binds damaged DNA; Rad4p-Rad23p heterodimer binds to promoters of DNA damage response genes to repress their transcription in the absence of DNA damage	0.0188
RAD4*	−2.22	Protein that recognizes and binds damaged DNA (with Rad23p) during NER; subunit of Nuclear Excision Repair	0.0499
RAD1*^#^	−5.43	Single-stranded DNA endonuclease (with Rad10p); cleaves single-stranded DNA during nucleotide excision repair and double-strand break repair; subunit of Nucleotide Excision Repair Factor 1 (NEF1); homolog of human XPF protein	0.0612
RAD5*	−3.79	DNA helicase/Ubiquitin ligase; involved in error-free DNA damage tolerance (DDT), replication fork regression during postreplication repair by template switching, error-prone translesion synthesis	0.0696
*CSM2^#^*	−1.25	Component of Shu complex (aka PCSS complex); Shu complex also includes Psy3, Shu1, Shu2, and promotes error-free DNA repair	0.0790
*PSY3*	−2.12	Component of Shu complex (aka PCSS complex); Shu complex also includes Shu1, Csm2, Shu2, and promotes error-free DNA repair; promotes Rad51p filament assembly	0.0926

a Genes in “bold” are those that are responsive to replication stress. *Appears twice among screens (q < 0.1). **Appears twice among screens (q < 0.1 and *P* < 0.05). *^#^*Transcription induced by AFB_1_ exposure.

b. m.value is the numeric vector of fold change on a log2 scale, rounded to three significant digits.

c Gene function descriptions obtained from www.yeastgenome.org.

d q value is the numeric vector calculated based on the p-value using the p.adjust function with default parameter settings, rounded to three significant digits.

**Figure 2:**
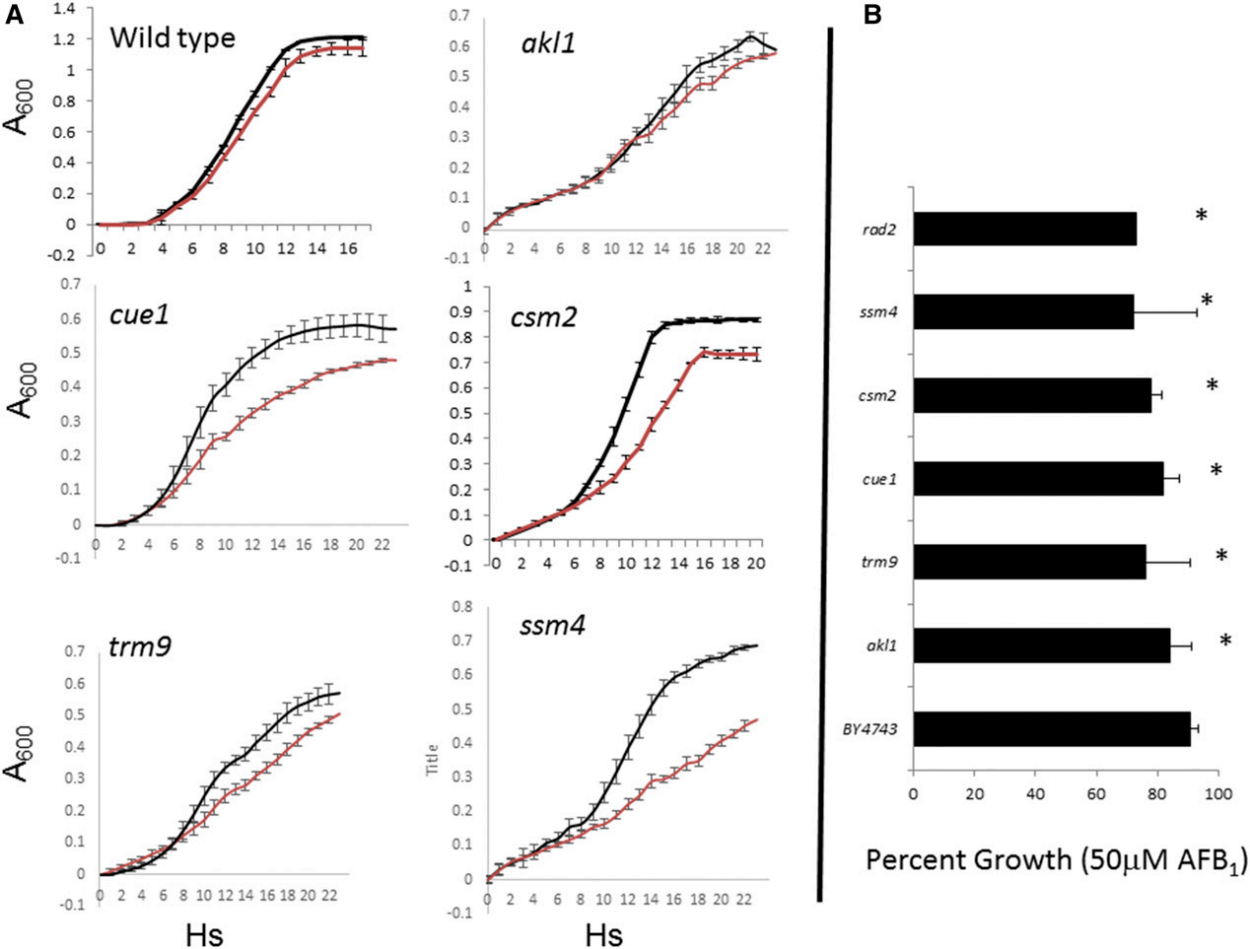
Growth curves for selected diploid mutants identified in the high throughput screen. All strains contain pCS316, expressing CYP1A2. A_600_ is plotted against time (Hs). Standard deviations are indicated at 1 h time points. The growth curves (Panel A) are indicated for wild type (YB556) and the *csm2*, *alk1*, *ssm4*, *cue1*, and *trm9* diploid mutants. Red lines indicate strains both expressing CYP1A2 and exposed to 50 μM AFB_1_. The bar graph (Panel B) indicates area percent growth of AFB_1_-exposed strains as determined by the ratio of the area under curves (area under the curve for treated strain/area under the curve for strain exposed to DMSO x 100%).

To confirm the AFB_1_-sensitivity for strains associated with negative m values, we determined percent growth inhibition for individual strains and compared calculations with that obtained for BY4743 expressing CYP1A2 (YB556). The percent growth inhibition for 86 strains after exposure to 50 μM AFB_1_ was determined are listed in Figure S1; significance was determined by comparison with YB556. Among strains that indicated both positive and negative m values, growth curves indicated that these deletion strains were actually more AFB_1_-sensitive compared to the wild-type BY4743, consistent with the negative m values. We confirmed that of 79 out of 86 strains (92%) are AFB_1_-sensitive (Figure S1); growth curves are shown for a subset of these strains (Figure 2) and trypan blue staining indicated that viability is lost among representative strains after acute AFB_1_ exposure (Figure S2). Strains which have not been confirmed by any criteria are listed in Table S5. AUCs were calculated for a few strains (*gtt1*, *ies2*, *sip18*, *tpo4*) that were only associated with positive m values; none of these were AFB_1_-sensitive.

Of the 79 AFB_1_-sensitive strains, 15 (19%) are deleted in well-documented DNA repair genes (Table 1). Another 64 are deleted for genes that have diverse functions (Table 2) but also include an additional five genes (*BLM10*, *FUM1*, *PSY2*, *DPB3*, *NUP60*) noted to confer resistance to diverse DNA damaging agents and placed in the DNA repair gene ontology group (Table 3). Five different genes, participating in nucleotide excision repair, postreplication repair and ribosome assembly were twice found among the 79 strains. An additional five genes were found that that were highly statistically different (q < 0.1) in one screen and statistically different in another screen (*P* < 0.05). Among these were those involved in proteolysis (*CUE1*), vacuolar acidification (*VOA1*), cell cycle progression (*FKH2*), DNA recombinational repair (*RAD55*) and postreplication repair *(**RAD18*).

**Table 2:** Fitness scores for 64 AFB_1_ resistant genes ranked by significance

Gene*^a^*	m. value*^b^*	Gene Function*^c^*	q.value*^d^*
*MIX23*	−4.06	Mitochondrial intermembrane space CX(n)C motif protein	4.35E-10
*MRPL35*	−4.28	Mitochondrial ribosomal protein of the large subunit	9.14E-10
*BIT2*	−3.96	Subunit of TORC2 membrane-associated complex	1.47E-08
*MNN10*	−4.30	Subunit of a Golgi mannosyltransferase complex	1.59E-08
*YND1*	−4.43	Yeast Nucleoside Diphosphatase	2E-08
*SPO1*	−3.52	Meiosis-specific prospore protein	5.48E-07
*PYK2*	−3.94	Pyruvate kinase; appears to be modulated by phosphorylation	8.44E-07
***TMA20***	−3.18	Protein of unknown function that associates with ribosomes; has a putative RNA binding domain; interacts with Tma22p; null mutant exhibits translation defects	9.88E-07
*DET1*	−4.35	Decreased ergosterol transport	2.06E-06
*TRX3*	−4.37	Mitochondrial thioredoxin	2.62E-06
*SSM4*	−1.67	Membrane-embedded ubiquitin-protein ligase; ER and inner nuclear membrane localized RING-CH domain E3 ligase involved in ER-associated protein degradation (ERAD)	5.11E-06
*AKL1*	−3.05	Ser-Thr protein kinase; member (with Ark1p and Prk1p) of the Ark kinase family; involved in endocytosis and actin cytoskeleton organization	5.26E-06
*PPG1*	−2.78	Putative serine/threonine protein phosphatase; putative phosphatase of the type 2A-like phosphatase family, required for glycogen accumulation	8.95E-05
***GTB1***	−2.68	Glucosidase II beta subunit, forms a complex with alpha subunit Rot2p; relocalizes from ER to cytoplasm upon DNA replication stress	8.95E-05
*NUP60*	−2.49	FG-nucleoporin component of central core of the nuclear pore complex; contributes directly to nucleocytoplasmic transport and maintenance of the nuclear pore complex (NPC) permeability barrier and is involved in gene tethering at the nuclear periphery; relocalizes to the cytosol in response to hypoxia	0.000118
*SVF1*	−2.61	Protein with a potential role in cell survival pathways; required for the diauxic growth shift; expression in mammalian cells increases survival under conditions inducing apoptosis	0.000253
*ATP15*	−3.46	Epsilon subunit of the F1 sector of mitochondrial F1F0 ATP synthase	0.000292
*ATO3*	−4.18	Plasma membrane protein, putative ammonium transporter	0.000931
*PET10*	−7.89	Protein of unknown function that localizes to lipid particles; large-scale protein-protein interaction data suggests a role in ATP/ADP exchange	0.00128
*DAL82*	−7.08	Positive regulator involved in the degradation of allantoin	0.00347
*PIB2*	−5.64	PhosphatidylInositol(3)-phosphate Binding	0.00421
*DPB3*	−2.16	Third-largest subunit of DNA polymerase II (DNA polymerase epsilon); required to maintain fidelity of chromosomal replication; stabilizes the interaction of Pol epsilon with primer-template DNA	0.00449
*CKB1*	−6.44	Beta regulatory subunit of casein kinase 2 (CK2); a Ser/Thr protein kinase with roles in cell growth and proliferation; CK2, comprised of CKA1, CKA2, CKB1 and CKB2, has many substrates including transcription factors and all RNA polymerases	0.00449
*RAV1*	−5.20	Regulator of (H+)-ATPase in Vacuolar membrane	0.00449
*SWM1^#^*	−2.14	Subunit of the anaphase-promoting complex (APC); APC is an E3 ubiquitin ligase that regulates the metaphase-anaphase transition and exit from mitosis	0.00449
*GRE3*	−2.09	Aldose reductase; involved in methylglyoxal, d-xylose, arabinose, and galactose metabolism; stress induced (osmotic, ionic, oxidative, heat shock, starvation and heavy metals)	0.00449
*HXK2*	−2.08	Hexokinase isoenzyme 2; phosphorylates glucose in cytosol; predominant hexokinase during growth on glucose; represses expression of HXK1, GLK1	0.00449
*PSY2*	−5.28	Subunit of protein phosphatase PP4 complex; Pph3p and Psy2p form the active complex; regulates recovery from the DNA damage checkpoint,; putative homolog of mammalian R3	0.00663
*DIT1*	−3.58	Sporulation-specific enzyme required for spore wall maturation	0.00676
*CKB2*	−2.15	Beta’ regulatory subunit of casein kinase 2 (CK2); a Ser/Thr protein kinase with roles in cell growth and proliferation	0.0103
*MIS1*	−1.72	Mitochondrial C1-tetrahydrofolate synthase; involved in interconversion between different oxidation states of tetrahydrofolate (THF); provides activities of formyl-THF synthetase, methenyl-THF cyclohydrolase, and methylene-THF dehydrogenase	0.0164
*ATP11*	−1.87	Molecular chaperone; required for the assembly of alpha and beta subunits into the F1 sector of mitochondrial F1F0 ATP synthase	0.0193
*SCW10*	−7.88	Cell wall protein	0.0193
*ROT2*	−1.74	Glucosidase II catalytic subunit; required to trim the final glucose in N-linked glycans; required for normal cell wall synthesis	0.0200
*FKH2*	−1.70	Forkhead family transcription factor; rate-limiting activator of replication origins	0.0204
*TRM9*	−2.32	tRNA methyltransferase; catalyzes modification of wobble bases in tRNA anticodons to 2, 5-methoxycarbonylmethyluridine and 5-methoxycarbonylmethyl-2-thiouridine; may act as part of a complex with Trm112p	0.0228
*HHF1*	−1.78	Histone H4	0.0265
***ATG29***	−2.10	Autophagy-specific protein; required for recruiting other ATG proteins to the pre-autophagosomal structure (PAS)	0.0265
*MYO4*	−1.71	Type V myosin motor involved in actin-based transport of cargos	0.0265
*PEX3*	−2.01	Peroxisomal membrane protein (PMP); required for proper localization and stability of PMP	0.0338
*FUM1*	−4.56	Fumarase; converts fumaric acid to L-malic acid in the TCA cycle	0.0338
*NRP1*	−1.48	Putative RNA binding protein of unknown function; localizes to stress granules induced by glucose deprivation; predicted to be involved in ribosome biogenesis	0.0340
CUE1**^#^	−1.09	Ubiquitin-binding protein; ER membrane protein that recruits and integrates the ubiquitin-conjugating enzyme Ubc7p into ER membrane-bound ubiquitin ligase complexes that function in the ER-associated degradation (ERAD) pathway for misfolded proteins	0.0378
*YIH1*	−1.74	Negative regulator of eIF2 kinase Gcn2p	0.0403
*GLO1*	−1.84	Monomeric glyoxalase I; catalyzes the detoxification of methylglyoxal (a by-product of glycolysis) via condensation with glutathione to produce S-D-lactoylglutathione; expression regulated by methylglyoxal levels and osmotic stres	0.0473
*CLB5*	−1.74	B-type cyclin involved in DNA replication during S phase	0.0475
VOA1**	−6.42	ER protein that functions in assembly of the V0 sector of V-ATPase; functions with other assembly factors; null mutation enhances the vacuolar ATPase (V-ATPase) deficiency of a vma21 mutant impaired in endoplasmic reticulum (ER) retrieval	0.0499
*RPN10*	−1.88	Non-ATPase base subunit of the 19S RP of the 26S proteasome	0.0537
*RPS4A*	−1.66	Protein component of the small (40S) ribosomal subunit; mutation affects 20S pre-rRNA processing; homologous to mammalian ribosomal protein S4	0.0549
*RNR3*	−1.37	Minor isoform of large subunit of ribonucleotide-diphosphate reductase; the RNR complex catalyzes rate-limiting step in dNTP synthesis, regulated by DNA replication and DNA damage checkpoint pathways via localization of small subunit	0.0563
*DST1^#^*	−1.88	General transcription elongation factor TFIIS; enables RNA polymerase II to read through blocks to elongation by stimulating cleavage of nascent transcripts stalled at transcription arrest sites	0.0567
*BCK2*	−1.76	Serine/threonine-rich protein involved in PKC1 signaling pathway; protein kinase C (PKC1) signaling pathway controls cell integrity; overproduction suppresses pkc1 mutation	0.0696
*BLM10*	−1.52	Proteasome activator; binds the core proteasome (CP) and stimulates proteasome-mediated protein degradation by inducing gate opening; required for sequestering CP into proteasome storage granule (PSG) during quiescent phase	0.0718
*ERV46*	−1.34	Protein localized to COPII-coated vesicles; forms a complex with Erv41p; involved in the membrane fusion stage of transport	0.0779
*AUA1*	−2.33	Protein required for the negative regulation by ammonia of Gap1p; Gap1p is a general amino acid permease	0.0779
*DUS1*	−1.81	Dihydrouridine synthase; member of a widespread family of conserved proteins including Smm1p, Dus3p, and Dus4p; modifies pre-tRNA(Phe) at U17	0.0785
*RIT1*	−0.915	Initiator methionine 2’-O-ribosyl phosphate transferase; modifies the initiator methionine tRNA at position 64 to distinguish it from elongator methionine tRNA	0.0807
***GFD1***	−7.71	Coiled-coiled protein of unknown function; identified as a high-copy suppressor of a dbp5 mutation; protein abundance increases in response to DNA replication stress	0.0846
BUD20*	−1.08	C2H2-type zinc finger protein required for ribosome assembly; shuttling factor which associates with pre-60S particles in the nucleus, accompanying them to the cytoplasm	0.0849
*LAG2*	−1.52	Protein that negatively regulates the SCF E3-ubiquitin ligase; regulates by interacting with and preventing neddyation of the cullin subunit, Cdc53p	0.0899
*CLG1*	−5.33	Cyclin-like protein that interacts with Pho85p; has sequence similarity to G1 cyclins PCL1 and PCL2	0.0899
*MET6*	−5.90	Cobalamin-independent methionine synthase; involved in methionine biosynthesis and regeneration; requires a minimum of two glutamates on the methyltetrahydrofolate substrate	0.0917
*RVS167*	−3.59	Calmodulin-binding actin-associated protein; roles in endocytic membrane tabulation and constriction, and exocytosis	0.0926
*BSC1*	−2.24	Protein of unconfirmed function; similar to cell surface flocculin Flo11p;	0.0932

a Genes in “bold” are those that are responsive to replication stress. *Appears twice among screens (q < 0.1). **Appears twice among screens (q < 0.1 and *P* < 0.05). *^#^*Transcription induced by AFB_1_ exposure.

b. m.value is the numeric vector of fold change on a log2 scale, rounded to three significant digits.

c Gene function descriptions obtained from www.yeastgenome.org.

d q value is the numeric vector calculated based on the p-value using the p.adjust function with default parameter settings, rounded to three significant digits.

**Table 3:** Aflatoxin resistant genes categorized by gene ontology groups involved in biological process and function

Term_ID	Description	P value*^a^*	Annotations*^b^*	Annotated Genes
Process GO:0006974	Cellular response to DNA damage stimulus	9.872E-09	22	*RAD4*, *CSM2*, *RAD23*, *RAD54*, *MMS4*, *DPB3*, *RAD55*, *RAD1*, *REV1*, *RAD18*, *CKB2*, *PSY3*, *REV3*, *FUM1*, *CKB1*, *BLM10*, *RAD2*, *RAD10*, *RAD17*, *NUP60*, *RAD5*, *PSY2*
GO:0006281	DNA repair	3.47E-08	20	*RAD4*, *CSM2*, *RAD23*, *RAD54*, *MMS4*, *DPB3*, *RAD55*, *REV1*, *RAD1*, *RAD18*, *PSY3*, *REV3*, *FUM1*, *BLM10*, *RAD2*, *RAD10*, *RAD17*, *NUP60*, *RAD5*, *PSY2*
GO:0019985	Translesion synthesis	2.56E-07	7	*CSM2*, *REV1*, *RAD5*, *DPB3*, *RAD18*, *PSY3*, *REV3*
GO:0000731	DNA synthesis involved in DNA repair	6.13E-07	7	*CSM2*, *REV1*, *RAD5*, *DPB3*, *RAD18*, *PSY3*, *REV3*
GO:0070987	Error-free translesion synthesis	7.62E-07	6	*CSM2*, *REV1*, *RAD5*, *RAD18*, *PSY3*, *REV3*
GO:0006259	DNA metabolic process	6.97E-06	23	*RAD4*, *FKH2*, *CSM2*, *RAD23*, *RAD54*, *MMS4*, *DPB3*, *RAD55*, *RAD1*, *REV1*, *RNR3*, *RAD18*, *PSY3*, *REV3*, *FUM1*, *CLB5*, *BLM10*, *RAD2*, *RAD10*, *RAD17*, *NUP60*, *RAD5*, *PSY2*
GO:0006301	Postreplication repair	1.47-05	7	*CSM2*, *REV1*, *RAD5*, *DPB3*, *RAD18*, *PSY3*, *REV3*
GO:0051716	Cellular response to stimulus	1.93E-05	34	*RAD4*, *GRE3*, *PSY3*, *SVF1*, *RAD2*, *MRPL35*, *RAD10*, *BIT2*, *NUP60*, *RAD5*, *DAL82*, *TRX3*, *PSY2*, *CUE1*, *CSM2*, *MMS4*, *RAD54*, *HXK2*, *RAD23*, *DPB3*, *YIH1*, *TRM9*, *RAD55*, *REV1*, *RAD1*, *TCM62*, *SSM4*, *RAD18*, *CKB2*, *REV3*, *FUM1*, *CKB1*, *BLM10*, *RAD17*
GO:0006950	Response to stress	2.03E-05	29	*RAD4*, *CUE1*, *CSM2*, *RAD23*, *RAD54*, *MMS4*, *YIH1*, *DPB3*, *GRE3*, *RAD55*, *RAD1*, *REV1*, *TCM62*, *SSM4*, *CKB2*, *RAD18*, *PSY3*, *FUM1*, *REV3*, *SVF1*, *CKB1*, *BLM10*, *RAD2*, *RAD10*, *RAD17*, *NUP60*, *RAD5*, *TRX3*, *PSY2*
GO:0006302	Double-strand break repair	2.05E-05	12	*RAD1*, *PSY3*, *REV3*, *RAD10*, *CSM2*, *RAD17*, *NUP60*, *RAD5*, *RAD54*, *MMS4*, *PSY2*, *RAD55*
GO:0042276	Error-prone translesion synthesis	3.69E-05	6	*REV1*, *RAD5*, *RAD18*, *DPB3*, *REV3*
GO:1903046	Meiotic cell cycle process	0.00490	13	*RAD1*, *DIT1*, *HHF1*, *CLB5*, *SPO1*, *RAD10*, *CSM2*, *RAD17*, *RAD54*, *MMS4*, *SWM1*, *PSY2*, *RAD55*
GO:0071897	DNA biosynthetic process	0.00210	9	*RAD1*, *REV1*, *RAD18*, *PSY3*, *REV3*, *RAD10*, *CSM2*, *RAD5*, *DPB3*
Function GO:0003684	Damaged DNA binding	4.50e-05	6	*RAD1*, *RAD17*, *REV1*, *RAD4*, *RAD23*, *RAD10*
GO:0004536	Deoxyribonuclease activity	0.00759	5	*RAD1*, *DPB3*, *RAD10*, *RAD2*, *RAD55*

a Adjusted P value using Bonferroni Correction, rounded to three significant digits.

b. Total annotated genes out of 79 genes.

According to GO process enrichment (https://go.princeton.edu/cgi-bin/GOTermFinder), resistance genes included those that function in the DNA damage response, DNA repair, postreplication repair, DNA damage stimulus, and meiotic cell cycle progression; 13 GO groups are shown in Table 3 (for full list of complete 86 putative ORFs, see Table S6). Of the 79 AFB_1_ resistance genes, 42 genes belong to the top 13 GO groups. One GO group that was unexpected was meiotic cell cycle process, which includes meiotic-specific genes *SPO1* and *DIT1*. Among the stress responsive genes are those that function in cell wall maintenance and glycogen metabolism, which were previously identified to confer resistance to a variety of toxins, such as benzopyrene and mycophenolic acid (O’Connor *et al.* 2012). Other genes involved in carbohydrate metabolism, such as *GRE3* that encodes aldose reductase, could have a direct role in detoxification and is induced by cell stress (Barski *et al.* 2008). Genes involved in rearrangement of the cellular architecture include *BIT2*, *AKL1* and *PPG1*; these genes function to rearrange the cellular architecture when cells are stressed (Schmidt *et al.* 1996). Thus, among AFB_1_ resistance genes are those that function to maintain structural integrity by affecting the cytoskeletal and cell wall architecture.

Other gene ontology groups encompass functions involved in mitochondrial maintenance and response to oxidative stress, and RNA metabolism (Table S6). Genes involved in mitochondrial function and response to oxidative stress include *TRX3*, *MRPL35*, *MIX23*, *MIS1*; *TRX3* (thioredoxin reductase) functions to reduce oxidative stress in the mitochondria (Greetham and Grant 2009). RNA metabolism genes include those involved in chemical modification of tRNA, including *MIS1*, *TRM9*, *DUS1*, and *RIT1*, and those involved in RNA translation, such as *TMA20* and *YIH1*. *TRM9* confers resistance to alkylated DNA damage, and links translation with the DNA damage response (Begley *et al.* 2007). These genes are consistent with the notion that AFB_1_ causes oxidative damage and that mitochondria are targets of AFB_1_-induced DNA damage.

In grouping genes according to protein function and cellular components (Table 3 and Table 4), DNA repair complexes were readily identified. Among these were the Shu complex, and the NER complexes I and II (Table 4). However, other interesting complexes that were identified included the glycosidase II complex, and the CK2 complex. Because DNA repair and DNA damage response genes were the most prominent of the GO groups, we focused on the function of these genes in conferring AFB_1_ resistance. As expected, these genes included those that participate in DNA recombination (*RAD54*, *RAD55*), nucleotide excision repair (*RAD1*, *RAD4*, *RAD1**,**RAD10*, *RAD23*), and postreplication repair (*RAD5*, *RAD18*, *REV1*, *REV3*). Many of these genes function in cell cycle progression. For example, *PSY2* and *CKB2* (Toczyski *et al.* 1997) promote cell cycle progression after cell cycle delay or arrest caused by stalled forks or double-strand breaks, respectively.

**Table 4:** Protein complexes that participate in AFB_1_ resistance

GO-term	Description*^a^*	Count in gene set*^b^*	Genes*^c^*	False discovery rate
GO:1990391	DNA repair complex	7 of 28	*RAD1*, *RAD2 RAD10*, *RAD4*, *RAD23*, *CSM2*, *PSY3*	5.22E-05
GO:0000109	Nucleotide-excision repair complex	5 of 16	*RAD1*, *RAD2 RAD10*, *RAD4*, *RAD23*	0.00063
GO:0017177	Glucosidase II complex	2 of 2	*GTB1*, *ROT2*	0.0214
GO:0000111	Nucleotide-excision repair factor 2 complex	2 of 3	*RAD4*, *RAD23*	0.0299
GO:0000110	Nucleotide-excision repair factor 1 complex	2 of 3	*RAD1*, *RAD10*	0.02909
GO:0005956	Protein kinase CK2 complex	2 of 4	*CKB1*, *CKB2*	0.0385
GO:0097196	Shu complex	2 of 4	*CSM2*, *PSY3*	0.0385

a See https://string-db.org/ and https://go.princeton.edu/

b Represents the number present among 79 genes.

c See Table 1 and Table 2 for full description.

**Figure 3:**
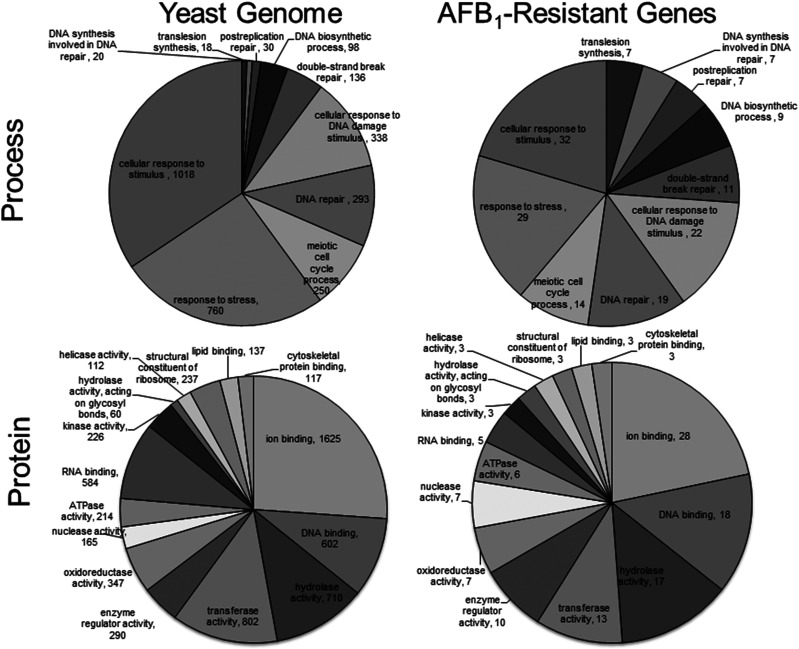
GO enrichment 79 AFB_1_ resistance genes, according to biological process and protein function. The top circles represent the number of genes of that are grouped according to Process, using the Generic Gene Ontology Term Finder, http://go.princeton.edu/cgibin/GOTermFinder. The bottom circles are those which are grouped according to protein function, using GO term finder according to function, https://www.yeastgenome.org/goSlimMapper. GO process groups included translesion synthesis, DNA synthesis involved in DNA repair, postreplication repair, DNA biosynthetic process, cellular response to DNA damage, DNA repair, meiotic cell cycle process, response to stress, and cellular response to stimulus. GO function groups include ion binding, hydrolase, nucleic acid binding, oxidoreductase, and transcription factors. Number of genes belonging to each GO is indicated within or just outside the pie slice.

To determine which GO biological processes and protein functions were most enriched among the AFB_1_ resistance genes we used the Panther software (Mi *et al.* 2016). A larger proportion of the AFB_1_ resistance genes are involved in DNA repair and metabolism, compared with the genome at large (Figure 3). A fraction of these genes also participate in postreplication repair, including DNA tolerance pathways that are error-free and error-prone replication (Table 5). In classifying protein functions, we analyzed whether hydrolases, nucleases, phosphatases, DNA damage binding were more enriched among the 79 AFB_1_ resistance genes, compared to the genome, at large (Figure 3). Of these groups, DNA damage binding was enriched among resistance genes (*P* < 0.05).

**Table 5:** Enrichment of DNA repair and stress-responsive genes among AFB_1_ resistant genes

GO Biological Process	Yeast*^a^*	AFB_1_ Resistant Genes	Expected*^b^*	Fold*^c^* Enrichment	*P* value*^d^*^.^	Significance*^e^*^.^
Cellular response to DNA damage stimulus	338	22	5	4.5	1.20E-06	+
DNA synthesis involved in DNA repair	20	7	<1	>7	8.00E-05	+
Translesion synthesis	18	7	<1	>7	4.42E-05	+
DNA repair	293	19	4	4.5	2.47E-05	+
Cellular response to stress	711	29	9.4	3.1	1.48E-04	+
Postreplication repair	30	7	<1	>7	8.35E-04	+
Double-strand break repair	136	11	2	5.6	5.97 E-03	+
DNA biosynthetic process	98	9	1.4	6.4	1.58 E-02	+

a Number of total yeast genes in GO group based on reference list of 6026.

b Number of expected genes among initial set of 79 ORFs based on reference list of 6026 genes; fractional values less than 1 were designated as <1.

c Fold enrichment is the ratio of the number of AFB1 resistant genes identified to the expected number.

d Displaying only results for Bonferroni-corrected probabilities, *P* < 0.05.

e Significance indicated by “+”

**Figure 4:**
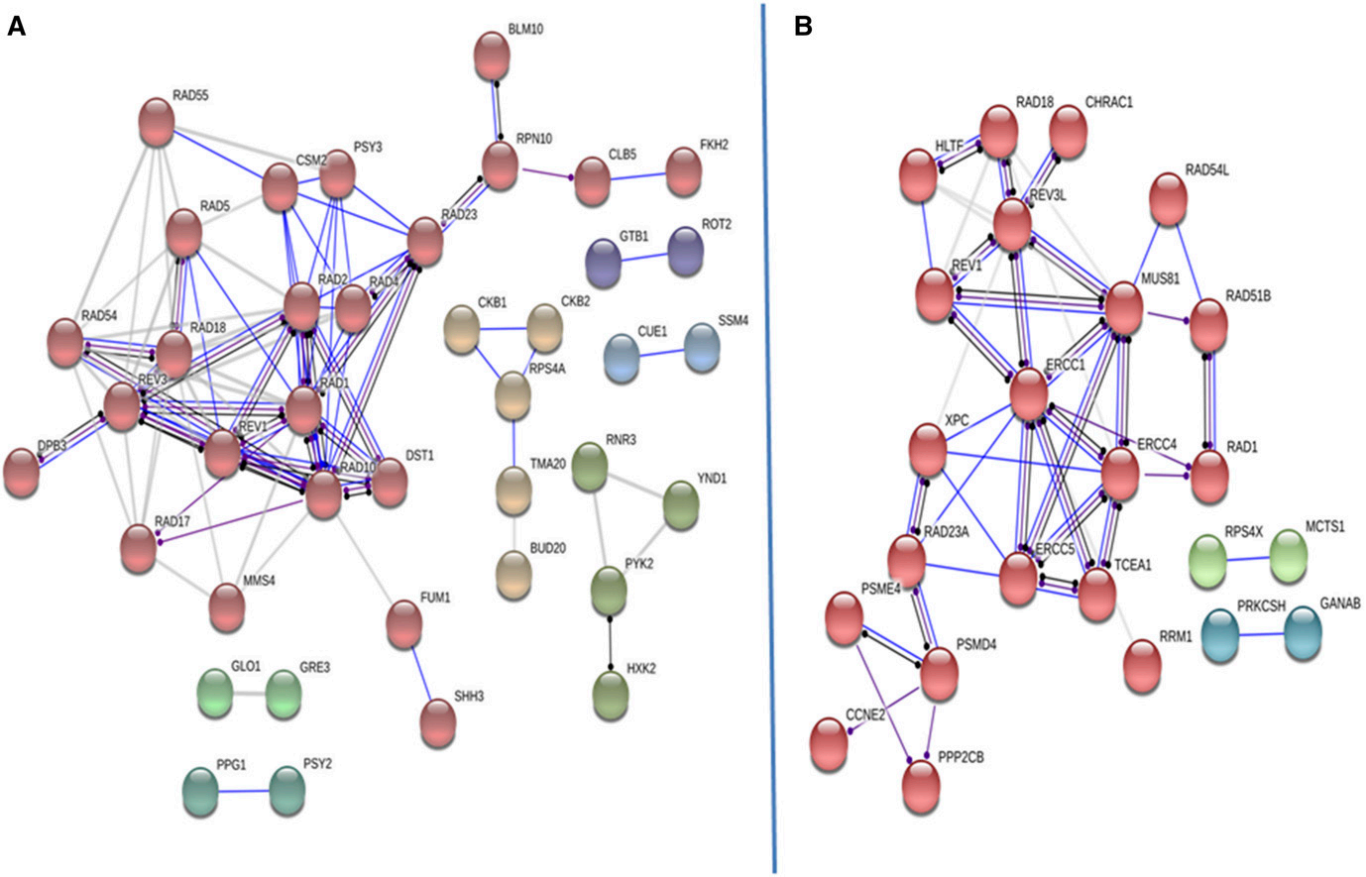
The protein interactome encoded by AFB_1_ resistance genes in budding yeast (left, A) and protein interactome encoded by their associated human homologs (right, B). The interactome was curated using String V11 (https://string-db.org, Szklarczyk *et al.* 2019), using a high confidence level of 0.7 and MCL cluster factor of 1.1. Proteins are represented by colored circles (nodes); different colors represent distinct interacting clusters. A core group, in red seen in both images, includes proteins that function in DNA repair pathways, and interact with proteases, transcription and cell cycle factors. Lines represent the edges; a solid blue line indicates a binding event, a dark line indicates a reaction, and a purple line indicates catalysis. The lighter lines indicate a strong connection, as deduced from the literature. Lines that terminate with a dot indicate an unspecified interaction, whether positive or negative.

To further determine the strength of the interactions among the confirmed 79 AFB_1_ resistance genes, we performed interactome mapping, using STRING software (https://string-db.org/, Szklarczyk *et al.* 2019), which associates proteins according to binding, catalysis, literature-based, and unspecified interactions (Figure 4). The interactome complex in yeast included 78 nodes and 152 edges with a 3.77 average node degree. Besides the NER complexes, individual complexes included the Shu complex, the Glucosidase II complex, and the protein kinase CK2 complex (Table 4); the glucosidase II complex is conserved in mammalian cells (Figure 4). While the strength and number of these interactions was particularly strong among the DNA repair genes, other interactions were elucidated, such as the interactions of protease proteins with cell cycle transcription factors and cyclins (Figure 4).

Since many known AFB_1_ resistance genes, which function in DNA repair and postreplication repair pathways, were not present among highly statistically different genes (q < 0.1), we also used a less stringent (*P* < 0.05) qualifier to identify potential AFB_1_ resistance genes. Among genes identified were additional members of the SHU complex, including *SHU1* and *SHU2*. These genes were confirmed by additional growth curves (Figure S3) and the percent growth was determined (Figure S1).

**Figure 5:**
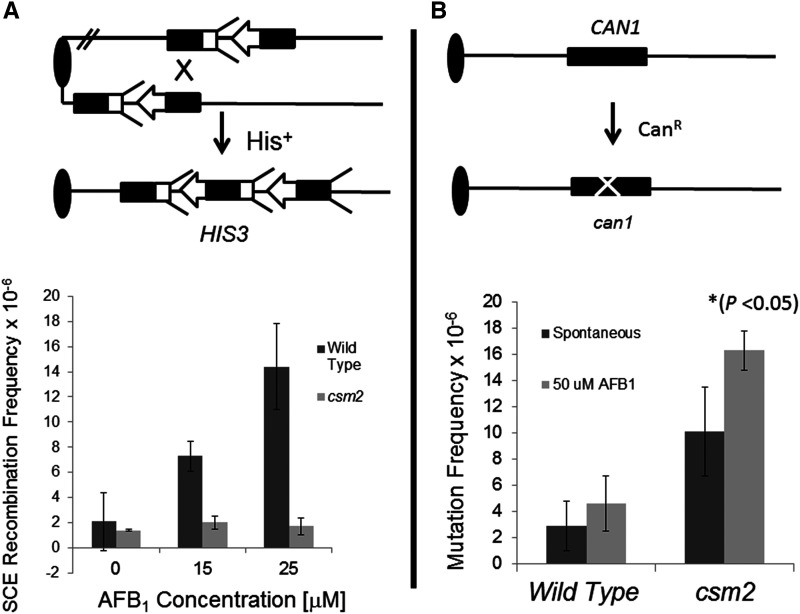
AFB_1_-associated sister chromatid recombination and mutagenesis frequencies in the wild type and *csm2* haploid mutant. The top part of each panel shows the assays for sister chromatid exchange and mutagenesis; for both assays the oval represents the centromere and the single line represents duplex DNA. For simplicity, the left arm of chromosomes IV and V are not shown. (Top left, A) Unequal sister chromatid recombination is monitored by selecting for His^+^ prototrophs that result from recombination between the juxtaposed, truncated *his3* fragments. The *his3*-Δ*3′* lacks the 3′ sequences (arrow head), while the *his3*-Δ*5′* lacks to promoter sequences (feathers). Both *his3* fragments are located with the amino acid reading frames oriented to the centromere. The *his3* fragments share a total of 450 bp sequence homology. Bottom of panel A shows the frequencies of unequal sister chromatid exchange (SCE) obtained from the wild type (YB204 pCS316) and the haploid *csm2* mutant (YB559) after exposure to 0, 15, and 25 μM AFB_1_. (Top right, B) The arrow notes the occurrence of point, missense, or deletion mutations that can occur in the *CAN1* gene and result in canavanine resistance (Can^R^) mutations. Bottom of panel B shows the frequencies of (Can^R^) mutants in wild type (YB204 pCS316) and *csm2* (YB559) strain after exposure to 0 and 50 μM AFB_1_. For complete genotype, see Table S1.

The SHU complex was previously identified as participating in error-free DNA damage tolerance and mutation avoidance (Shor *et al.* 2005, Xu *et al.* 2013). The complex confers resistance to alkylating agents, such as methyl methanesulfonate (MMS), and cross-linking agents, such as cisplatin, but not to UV and X-ray (Godin *et al.* 2016). We previously showed that while X-ray associated unequal SCE (SCE) was *RAD5*-independent (Fasullo and Sun 2017), MMS and 4NQO-associated unequal SCE occurs by well-conserved *RAD5*-dependent mechanisms (Unk *et al.* 2010; Fasullo and Sun 2017). We therefore postulated that the SHU complex suppresses AFB_1_-associated mutagenesis while promoting AFB_1_-associated template switching. We introduced pCS316 (CYP1A2) into both the haploid wild-type strain (YB204) and a *csm2* mutant (YB558, see Table S1) to measure frequencies of AFB_1_-associated unequal SCE and *can1* mutations. Our results showed that while we observed a threefold increase in SCE after exposure to AFB_1_ in wild type strains, we observed less than a twofold increase in sister chromatid exchange in the *csm2* mutant (Figure 5). However, we observed a net increase in AFB_1_-associated Can^R^ mutations in the in *csm2* mutant, compared to wild type (*P* < 0.05). Average survival was only slightly higher in the wild type (51%) than in the *csm2* mutant (49%), but not statistically different (*P* = 0.8, N =4). We suggest that similar to MMS-associated DNA lesions, *CSM2* functions to suppress AFB_1_-associated mutagenesis while promoting template switching of AFB_1_-associated DNA adducts.

**Figure 6:**
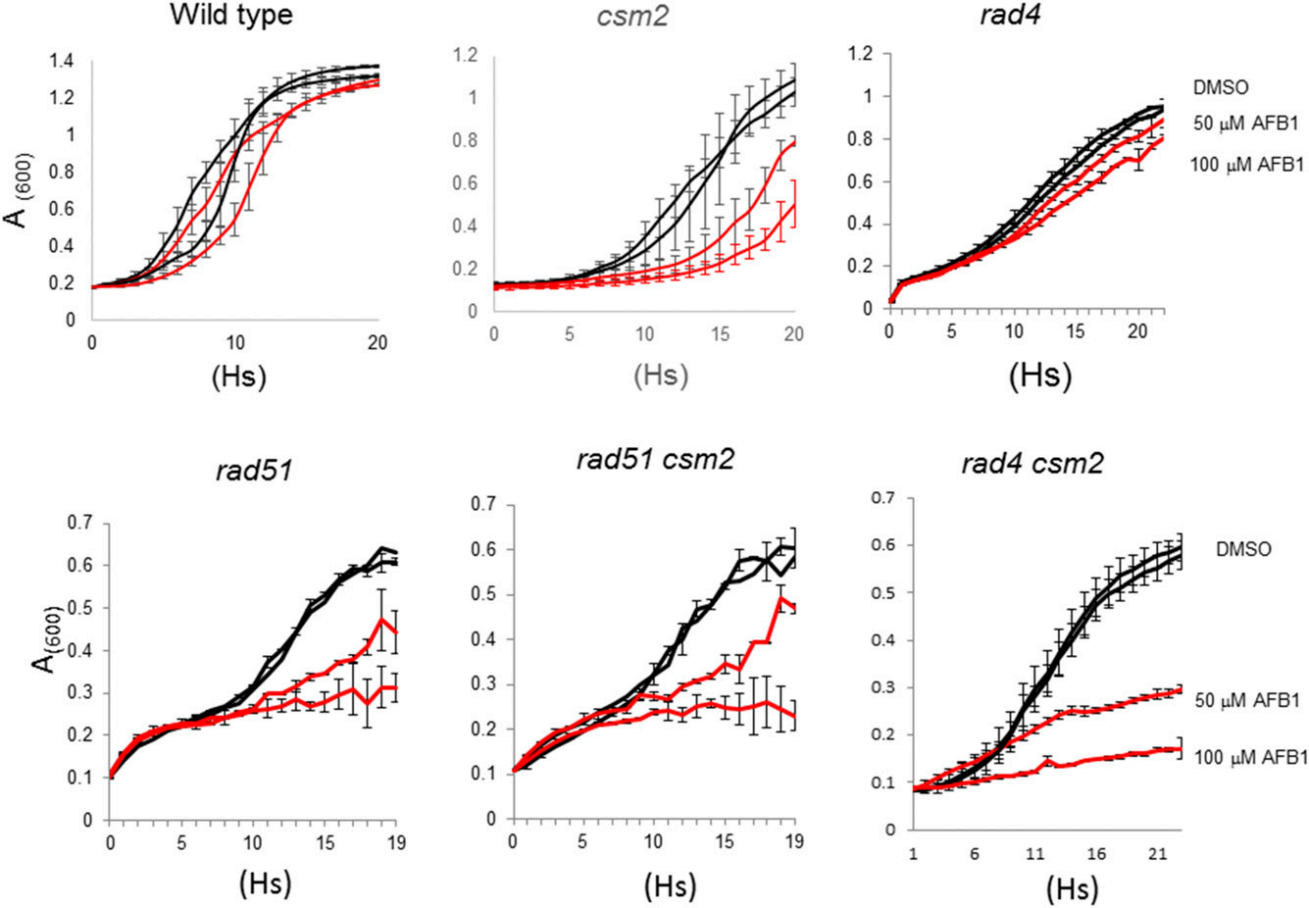
Growth curves of wild type (YB155), *csm2* (YB557), *rad4* (YB661), *rad51* (YB177), *csm2* *rad51* (YB663), and *csm2* *rad4* (YB660) haploid cells after exposure to 50 and 100 μM AFB_1_. (Left) Growth of cells containing pCS316 and expressing CYP1A2 after chronic exposure to 0.5% and 1.0% DMSO (black), 50 *μ*M (red), and 100 *μ*M (red) AFB_1_. The relevant genotype is given above the panel (see Table S1, for complete genotype). Approximately 10^5^ log-phase cells were inoculated in each well, n *=* 2. *A*_600_ is plotted against time (hs). Bars indicate the standard deviations of measurements, n = 2.

If *CSM2* participates in a *RAD51*-dependent recombinational repair pathway to tolerate AFB1-associated DNA lesions, then we would expect that *RAD51* would be epistatic to *CSM2* for AFB_1_ resistance (Glassner and Mortimer 1994). We measured AFB_1_ sensitivity in the *csm2*, *rad51* and *csm2* *rad51* haploid mutants compared to wild type, using growth curves (Figure 6). Our data indicate the *csm2* *rad51* double mutant is no more AFB_1_ sensitive compared to either *rad51* single mutants indicating that *CSM2* and *RAD51* are in the same epistasis group for AFB_1_ sensitivity. In contrast, *csm2* *rad4* double mutants are more sensitive to AFB_1_ than either the *csm2* and *rad4* single mutants; the fitness measurement of the double mutant (0.071) is also less than the product of the *csm2* (0.34) and *rad4* (0.28) single mutants. These data indicate that *CSM2* participates in a *RAD51*-mediated pathway for AFB_1_ resistance, and similar to *RAD51*, confers AFB_1_ resistance in a *rad4* mutant (Fasullo *et al.* 2010).

Human orthologs for many essential yeast genes can directly complement the corresponding yeast genes (Kachroo *et al.* 2015). Human homologs are listed for 46 yeast AFB_1_-resistance genes (Table 6). These homologs include those for DNA repair, DNA damage tolerance, cell cycle, and cell maintenance genes. Several of these genes, such as the human *CTR1* (Zhou and Gitschier 1997), can directly complement the yeast gene. Other DNA human DNA repair genes, such as those that encode *RAD54* (Kanaar *et al.* 1996), *RAD5* (Unk *et al.* 2010), and *RAD10* orthologs, can partially complement sensitivity to DNA damaging agents (Aggarwal and Brosh 2012).

**Table 6:** Human genes orthologous to yeast resistance genes

Yeast Gene*^a^*	Human Gene*^b^*	Description/Function
*AKL1*	AAK1	Adaptor protein 2 associated kinase 1
*ATP11*	ATPAF1	F1-ATPase assembly factor 1
*ATP15*	ATP5E	ϵ subunit of human ATP synthase
*BLM10*	PSME4	Proteasome activator complex subunit 4
*CKB1*	CSNK2B	Casein kinase II subunit beta
*CKB2*	CSNK2B	Casein kinase II subunit beta
*CLB5*	CCNE2	G1/S-specific cyclin-E2
*DST1*	TCEA1	Transcription elongation factor A1
*DPB3*	CHRAC1	Chromatin accessibility complex protein 1
*FKH2*	FOXE1	Forkhead box protein E1
*FUM1*	FH	Fumarate hydratase, mitochondrial
*GLO1*	GLO1	GLyOxalase
*GRE3*	AKRA1,B1,D1, E2, B10	Aldo-keto reductase family 1 member, A1, B1, D1, E2, B10
*GTB1*	PRKCSH	Protein kinase C substrate 80K-H
*HHF1*	HIST1H4D	Histone H4
*MIS1*	MTHFD1	Methylenetetrahydrofolate dehydrogenase
*MIX23*	CCDC58	Coiled-coil domain-containing protein 58
*MMS4*	EME1 and EME2	Essential meiotic structure-specific endonuclease 1, essential meiotic structure-specific endonuclease subunit 2
*MRPL35*	MRPL38	Large subunit mitochondrial ribosomal protein L38
*NRP1*	TEX13B	N (asparagine)-Rich Protein, Testis-expressed protein 13B
*PEX3*	PEX3	Peroxisomal biogenesis factor 3
*PPG1*	PPP2CB	Protein Phosphatase involved in Glycogen accumulation
*PSY2*	PPP4R3B	Serine/threonine-protein phosphatase 4 regulatory subunit 3B
*RAD1*	ERCC4	DNA repair endonuclease XPF
*RAD2*	ERCC5	DNA repair protein complementing XP-G cells
*RAD4*	XPC	DNA repair protein complementing XP-C cells
*RAD5*	HLTF	Helicase-like transcription factor involved in DNA damage tolerance
*RAD10*	ERCC1	Excision Repair Cross-Complementing 1 ERCC1
*RAD17*	RAD1	Cell cycle checkpoint protein RAD1
*RAD18*	RAD18	E3 ubiquitin-protein ligase RAD18
*RAD23*	RAD23A	UV excision repair protein RAD23 homolog A
*RAD54*	RAD54L	DNA repair and recombination protein RAD54-like
*RAD55*	RAD51B	DNA repair protein RAD51 paralog B
*RAV1*	DMXL2	Dmx like 2 also known as rabconnectin-3, involved in vacuolar acidification
*REV1*	REV1	REV1, DNA directed polymerase
*REV3*	REV3L	REV3 Like, DNA directed polymerase zeta catalytic subunit
*RNR3*	RRM1	Ribonucleotide reductase catalytic subunit M1
*ROT2*	GANAB	Glucosidase alpha, neutral C
*RPN10*	PSMD4	26S proteasome non-ATPase regulatory subunit 4
*RPS4A*	RPS4X	Ribosomal Protein of the Small subunit, 40S ribosomal protein S4, X isoform
*SPO1*	PLA2G4A	Phospholipase A2 group IVA
*SSM4*	MARCH6	Membrane associated ring-CH-type finger 6
*TMA20*	MCTS1	Malignant T-cell-amplified sequence 1, translation re-initiation and release factor
*TRM9*	ALKB8	Alkylated DNA repair protein alkB homolog 8
*YIH1*	IMPACT	Impact RWD domain protein**;** translational regulator that ensures constant high levels of translation upon a variety of stress conditions; impact RWD domain protein
*YND1*	ENTPD4 and ENTPD7	Ectonucleoside triphosphate diphosphohydrolase 4 and 7

a For full description, see Table 1 and Table 2.

b Human genes derived from https://www.alliancegenome.org/gene/SGD:S000004022.

## Discussion

Human CYP1A2-mediated activation of the mycotoxin AFB_1_ generates a highly reactive epoxide that interacts with DNA, RNA, and protein, forming adducts which interfere in replication, transcription, and protein function. Previous experiments have documented the role of checkpoint genes, RAD genes, and BER genes in conferring AFB_1_ resistance in budding yeast (Keller-Seitz *et al.* 2004; Guo *et al.* 2005; Fasullo *et al.* 2010). The goal of this project was to identify additional AFB_1_ resistance genes that may elucidate why AFB_1_ is a potent yeast recombinagen but a weak mutagen (Sengstag *et al.* 1996).

Here, we profiled the yeast genome for AFB_1_ resistance using three yeast non-essential diploid deletion libraries; one was the original library and the other two expressed human CYP1A2. We identified 96 ORFs, of which 86 have been ascribed a function and 79 were confirmed to be AFB_1_ sensitive, relative to the wild type. These resistance genes reflect the broad range of functions, including cellular and metabolic processes, actin reorganization, mitochondrial responses, and DNA repair. Many of the DNA repair genes and checkpoint genes have been previously identified in screens for resistance to other toxins (Lee *et al.* 2005; Giaever and Nislow 2014; De La Rosa *et al.* 2017). While individual resistance genes are shared among diverse toxins, such as doxorubicin, nystatin, cycloheximide, rapamycin, and amphotericin, the top ten AFB_1_-associated GO enrichments are not represented among these diverse toxins. However, AFB_1_-associated GO enrichments, including postreplication repair and translesion synthesis, are shared among genes that confer resistance to cross-linking agents, such as trichloroethylene (De La Rosa *et al.* 2017) and cisplatin (Lee *et al.* 2005). One similarity between cross-linking agents and metabolically activated AFB_1_ is that they can form DNA adducts that impede DNA replication.

While mitochondrial maintenance genes and oxidative stress genes (Amici *et al.* 2007; Mary *et al.* 2012) are expected AFB_1_ resistance genes based on studies of individual mutants (Guo *et al.* 2005; Fasullo *et al.* 2010), we also identified novel AFB_1_ resistance genes that participate in DNA postreplication repair, both by modulating checkpoint responses and by recombination-mediated mechanisms. Of key importance, the CSM2/SHU complex (Shor *et al.* 2005) was required for AFB_1_-associated sister chromatid recombination, underscoring the role of recombination-mediated template switch mechanisms for tolerating AFB_1_-associated DNA damage. Since many yeast genes are conserved in mammalian organisms (Bernstein *et al.* 2011), we suggest similar mechanisms for tolerating AFB_1_-associated DNA damage may be present in mammalian cells.

We used a novel reagent consisting of a pooled yeast library expressing human CYP1A2, on a multi-copied expression vector. Because CYP1A2 activates AFB_1_ when toxin concentration is low (Eaton and Gallagher 1994), our modified yeast library mimicked AFB_1_ activation when low hepatic AFB_1_ concentrations generate DNA adducts. One limitation of the screen in the CYP1A2-expressing library is that AFB_1_-associated toxicity is not directly proportional to AFB_1_ concentration (Fasullo *et al.* 2010); we speculate that CYP1A2 activity is the limiting factor. Although individual yeast strains expressed similar amounts of CYP1A2 activity from among the subset of deletion strains tested, it is still possible that profiling resistance among individual deletion strains is influenced by the stability or variable expression of the membrane-associated human CYP1A2 (Murray and Correia 2001). Second, many DNA repair and checkpoint genes that were previously documented to confer resistance, such as *RAD52*, were not identified in the screen (Guo *et al.* 2006; Fasullo *et al.* 2010). One possible reason is that some, such as *rad52*, grow poorly (Figure 1, Fasullo *et al.* 2008), and we suspect that other slow-growing strains dropped out early in the time course of exposure. Future experiments will more carefully assess generation times needed to detect known resistance genes in the library expressing CYP1A2.

Because metabolically activated AFB_1_ causes protein, RNA, and lipid damage (Weng *et al.* 2017), besides DNA damage, we expected to find a functionally diverse set of AFB_1_ resistance genes. Among AFB_1_ resistance genes were those involved in protein degradation and ammonia transport, actin reorganization, tRNA modifications, ribosome biogenesis, RNA translation, mitochondrial, and metabolic functions. Some genes encoding these functions, such as *BIT2* and *TRM9*, have important roles in maintaining genetic stability and in double-strand break repair (Schmidt *et al.* 1996; Begley *et al.* 2007; Schonbrun *et al.* 2013; Shimada *et al.* 2013). A more direct role in DNA repair mechanisms have been noted for *FUM1* (Leshets *et al.* 2018; Saatchi and Kirchmaier 2019), *DPB3* (Gallo *et al.* 2019), and *BLM10* (Qian *et al.* 2013). Several genes, such as *PPG1*, are involved in glycogen accumulation; these genes are also required to enter the quiescent state (Li *et al.* 2015). Other genes are involved in cell wall synthesis, including *MNN10*, *SCW10* and *ROT2*; we speculate that cell wall synthesis genes confer resistance by impeding AFB_1_ entrance into the cell, while genes involved in protein degradation in the ER may stabilize CYP1A2 and thus enhance AFB_1_-conferred genotoxicity. Indeed, two resistance genes, *CUE1* and *SSM4*, are associated with degradation of mammalian P450 proteins in yeast (Murray and Correia 2001). Glucan and other cell wall constituents have also been speculated to directly inactivate AFB1, and yeast fermentative products are supplemented in cattle feed to prophylactically reduce AFB1 toxicity (Pereyra *et al.* 2013).

Although AFB_1_-associated cellular damage is associated with oxidative stress (Amici *et al.* 2007; Mary *et al.* 2012; Liu and Wang 2016) only a few yeast genes that confer resistance to reactive oxygen species (ROS) were identified in our screens. These genes included *TRX3*, *YND1*, *VPS13*, *BIT2*, *GTP1*, *FKH2*, *SHH3*, *NRP1*, and *BUD20*, which have a wide variety of functions (Schmidt *et al.* 1996; Greetham and Grant 2009; Bassler *et al.* 2012). While known genes associated with oxidative stress and oxidative-associated DNA damage, such as *YAP1*, *SOD1*, and *APN1*, were not identified, other mitochondrial genes, such as *TRX3*, were identified. Guo *et al.* (2005) also showed that the haploid *apn1* mutant was not AFB_1_ sensitive. We offer two different explanations: first, the AFB_1_-associated oxidative damage is largely localized to the mitochondria, and second, there may be redundant pathways for conferring resistance to AFB_1_-associated oxidative damage, and therefore single genes were not identified. It is most likely the later as screens with oxidants (like t-BuOOH) also fail to identify expected antioxidant enzymes (Said *et al.* 2004).

A majority of the AFB_1_ resistance genes belong to GO groups that include postreplication repair, DNA damage-inducible genes, DNA repair, response to stimulus, or response to replication stress (Table 3). Proteins encoding functionally diverse genes, such as *RAD54*, *RAD5*, *GFD1*, *TMA20*, *SKG3*, *GRE3*, and *ATG29*, are repositioned in the yeast cells during DNA replication stress (Tkach *et al.* 2012). The requirement for unscheduled DNA synthesis was illustrated by identifying genes involved in the DNA damage-induced expression of ribonucleotide reductase; these included *RNR3* and *TRM9*. *TRM9*, involved in tRNA modification, functions to selectively translate DNA damage-inducible genes, such as *RNR1* (Begley *et al.* 2007).

One unifying theme was that cell cycle progression and recovery from checkpoint-mediated arrest is a prominent role in mediating toxin resistance. *FKH2* functions as a transcription factor that promotes cell cycle progression and G_2_-M progression. Other genes are involved in the modulation of the checkpoint response, such as *PSY2*. While *CKB1* and *CKB2* have broad functions, including histone phosphorylation and chromatin remodeling (Barz *et al.* 2003; Cheung *et al.* 2005), *CKB2* is also required for toleration of double-strand breaks (Guillemain *et al.* 2007; Toczyski *et al.* 1997), and thus may function for the toleration of AFB_1_-associated damage. These genes support the notion that some of the AFB_1_-associated DNA adducts are well tolerated and can be actively replicated.

While we expected to identify individual genes involved in DNA damage tolerance, such as *RAD5*, *REV1*, *REV3*, the *CSM2**/**PSY3* complex is novel. Absence of *CSM2* confers deficient AFB_1_-associated SCE but higher frequencies of AFB_1_-associated mutations, suggesting that *CSM2* functions to suppress AFB_1_-associated mutations by *RAD51*-mediated template switch mechanisms (Guo *et al.* 2006; Fasullo *et al.* 2008). Consistent with this idea, *RAD51* is epistatic to *CSM2* in conferring AFB_1_ sensitivity, while *rad4* *cms2* double mutant exhibits synergistic AFB_1_ sensitivity with respect to the single *rad4* and *csm2* single mutants. However, *rad4* *rad51* mutants still exhibit more AFB_1_ sensitivity than *rad4* *csm2*, suggesting that *RAD51* may be involved in conferring resistance to other AFB_1_-associated DNA lesions, such as double-strand breaks. We also expect that the *RAD51* paralogs, *RAD55* and *RAD57*, share similar AFB_1_-associated functions with *RAD51*. Considering that *RAD57* is the XRCC3 ortholog, determining whether yeast *RAD51* paralogs suppress AFB_1_-associated mutation will aid in identifying similar complexes in mammalian cells. Such complexes may elucidate why XRCC3 polymorphisms are risk factors in AFB_1_-associated liver cancer (Long *et al.* 2008; Ji *et al.* 2015).

Human homologs of several of the identified yeast genes (Table 6) are, hyper-methylated, mutated, or over-expressed in liver disease and cancer. For example, mutations and promoter methylations of the human *RAD5* ortholog, Helicase-Like Transcription Factor (HLTF), are observed in hepatocellular carcinoma (Zhang *et al.* 2013; Dhont *et al.* 2016). Mutations in PRKCSH and GANAB, human orthologs of *GTB1* and *ROT2*, are linked to polycystic liver disease (Porath *et al.* 2016; Perugorria and Banales 2017). The *CKB2* ortholog, CSNK2B (Zhang *et al.* 2015; Chua *et al.* 2017; Dotan *et al.* 2001), is over-expressed in several liver cancers and therapeutics are currently in clinical trial (Gray *et al.* 2014; Li *et al.* 2017; Trembley *et al.* 2017). It is tempting to speculate that over-expression of CSNK2B also confers AFB_1_ resistance.

Human homologs of other yeast genes have been correlated to the etiology and progression of other cancers, including colon and renal cancer. These include the human homolog for *TRM9*, ALKB8, and the human homolog for *TMA20*, MCT1, which can complement translation defects of *tma20* mutants and has been implicated in modulating stress (Herbert *et al.* 2001) and double-strand break repair (Hsu *et al.* 2007). MCT-1 overexpression and p53 is noted to lead to synergistic increases in chromosomal instability (Kasiappan *et al.* 2009). Heterozygous germline mutations of fumarate hydratase (FH) predispose for hereditary leiomyomatosis and renal cell carcinoma (Lehtonen *et al.* 2006). In both mammalian and yeast cells, FH participates in double-strand break repair (Leshets *et al.* 2018) and may thus suppress genetic instability.

In summary, we profiled the yeast genome for AFB_1_ resistance and identified novel genes that confer resistance. The novel genes included those involved in tRNA modifications, RNA translation, DNA repair, protein degradation, and actin reorganization. Genes that function in DNA damage response and DNA damage tolerance were over-represented, compared to the yeast genome. We suggest that the *CSM2* (SHU complex) functions to promote error-free replication of AFB_1_-asociated DNA damage, and it will be interesting to determine whether mammalian orthologs of the SHU complex function similarly.
